# The 1ALCTL and 1BLCTL isoforms of Arg/Abl2 induce fibroblast activation and extra cellular matrix remodelling differently

**DOI:** 10.1242/bio.038554

**Published:** 2019-03-05

**Authors:** Barbara Torsello, Sofia De Marco, Silvia Bombelli, Elisa Chisci, Valeria Cassina, Roberta Corti, Davide Bernasconi, Roberto Giovannoni, Cristina Bianchi, Roberto A. Perego

**Affiliations:** 1 School of Medicine & Surgery, University of Milano-Bicocca, 20900 Monza, Italy; 2 Department of Materials Science, University of Milano-Bicocca, 20125 Milan, Italy

**Keywords:** Arg, Abl2, Non-receptor tyrosine kinase, Fibroblast, Extra cellular matrix, Stroma remodelling

## Abstract

The fibrotic tissue and the stroma adjacent to cancer cells are characterised by the presence of activated fibroblasts (myofibroblasts) which play a role in creating a supportive tissue characterised by abundant extracellular matrix (ECM) secretion. The myofibroblasts remodel this tissue through secreted molecules and modulation of their cytoskeleton and specialized contractile structures. The non-receptor protein tyrosine kinase Arg (also called Abl2) has the unique ability to bind directly to the actin cytoskeleton, transducing diverse extracellular signals into cytoskeletal rearrangements. In this study we analysed the 1ALCTL and 1BLCTL Arg isoforms in Arg^−/−^ murine embryonal fibroblasts (MEF) cell line, focusing on their capacity to activate fibroblasts and to remodel ECM. The results obtained showed that Arg isoform 1BLCTL has a major role in proliferation, migration/invasion of MEF and in inducing a milieu able to modulate tumour cell morphology, while 1ALCTL isoform has a role in MEF adhesion maintaining active focal adhesions. On the whole, the presence of Arg in MEF supports the proliferation, activation, adhesion, ECM contraction and stiffness, while the absence of Arg affected these myofibroblast features.

This article has an associated First Person interview with the first author of the paper.

## Abstract (precis)

**Summary:** The non-receptor tyrosine kinase Arg and its isoforms modulate the extra cellular matrix production that is relevant in fibrosis and tumour growth, this may open future novel therapeutic approaches.

## INTRODUCTION

The fibrotic process, after a chronic injury, results in an excessive scar tissue deposition and development of fibrosis, and has in the activated fibroblasts (myofibroblasts) the main players (Yazdani et al., 2017). In fact, myofibroblasts secrete extra cellular matrix (ECM) proteins and also a wide range of cytokines promoting cell proliferation, migration, angiogenesis and recruitment of inflammatory cells (Mueller and Fusenig, 2004; Orimo et al., 2005). Fibroblast activation not only occurs as a result of chronic injury, but also during tumour growth that leads to tumour stroma formation. The stroma adjacent to cancer cells is a permissive and supportive environment (Mueller and Fusenig, 2004) in which there is a variable presence of myofibroblasts, also called cancer associated fibroblasts (CAF). Increasing attention has been focused on CAF because of their important role on tumour progression by the secretion of ECM and several cytokines. Myofibroblasts are also capable of remodelling ECM and acquiring specialised contractile features, which result in the reorganisation and contraction of ECM both in fibrotic and in tumour stroma. In particular, CAF promote matrix remodelling by soluble factors (Kalluri and Zeisberg, 2006) and by the generation of tracks that enable the collective invasion of the tumour cells. RhoGTPase activity in myofibroblasts is necessary for track generation (Gaggioli et al., 2007). Remodelling of the ECM and promotion of cancer cell invasion also requires myofibroblast cytoskeletal rearrangement through contraction of the actomyosin cytoskeleton assembled in stress fibres (Barron and Rowley, 2012; Calvo et al., 2013).

The non-receptor tyrosine kinase Arg (also called Abl2) (Kruh et al., 1990; Perego et al., 1991) shares with Abl1 the unique ability, among the tyrosine kinases, to bind directly to the cytoskeleton, transducing diverse extracellular signals into cytoskeletal rearrangements (Bradley and Koleske, 2009). In particular Arg, due to the presence in its sequence of two actin-binding domains, can bind and stabilise F-actin filaments preventing their cut by cofilin. Arg is able to fasciculate F-actin and both domains are necessary for actin bundling and cytoplasmic distribution of stress fibres (Miller et al., 2004). ARG gene, through alternative splicing events, codes eight different isoforms, based on the reciprocal presence or absence of exons IA, IB and II in 5′-ends and a ΔCT sequence, including part of one of the F-actin-binding domain, in 3′-ends of the specific Arg transcript (Bianchi et al., 2008). The eight different Arg isoforms are expressed in normal and neoplastic cells with different ratios (Perego et al., 2005). Overexpression of the different Arg isoforms in COS cells induces different effects on cell morphology and cytoskeleton organisation. In particular, 1ALCTL and 1BLCTL Arg isoforms, differing only in the 1A and 1B exons, determine a different behaviour of transfected COS cells. The 1BLCTL isoform induces a higher reduction of cell surface area and stress fibre density with respect to 1ALCTL isoform and enhances the formation of filopodia structures instead of lamellipodia and retraction tails (Bianchi et al., 2013).

The aim of this study was to analyse more deeply the difference of two 1ALCTL and 1BLCTL Arg isoforms transfected in Arg^−/−^ murine embryonal fibroblasts (MEF) cell line, in particular focusing on their capacity to activate fibroblasts, to modulate their functionality and to remodel ECM.

## RESULTS

### Stable expression and tyrosine kinase activity of 1ALCTL and 1BLCTL Arg isoforms transfected in Arg^−/−^ MEF

**Fig. 1.**
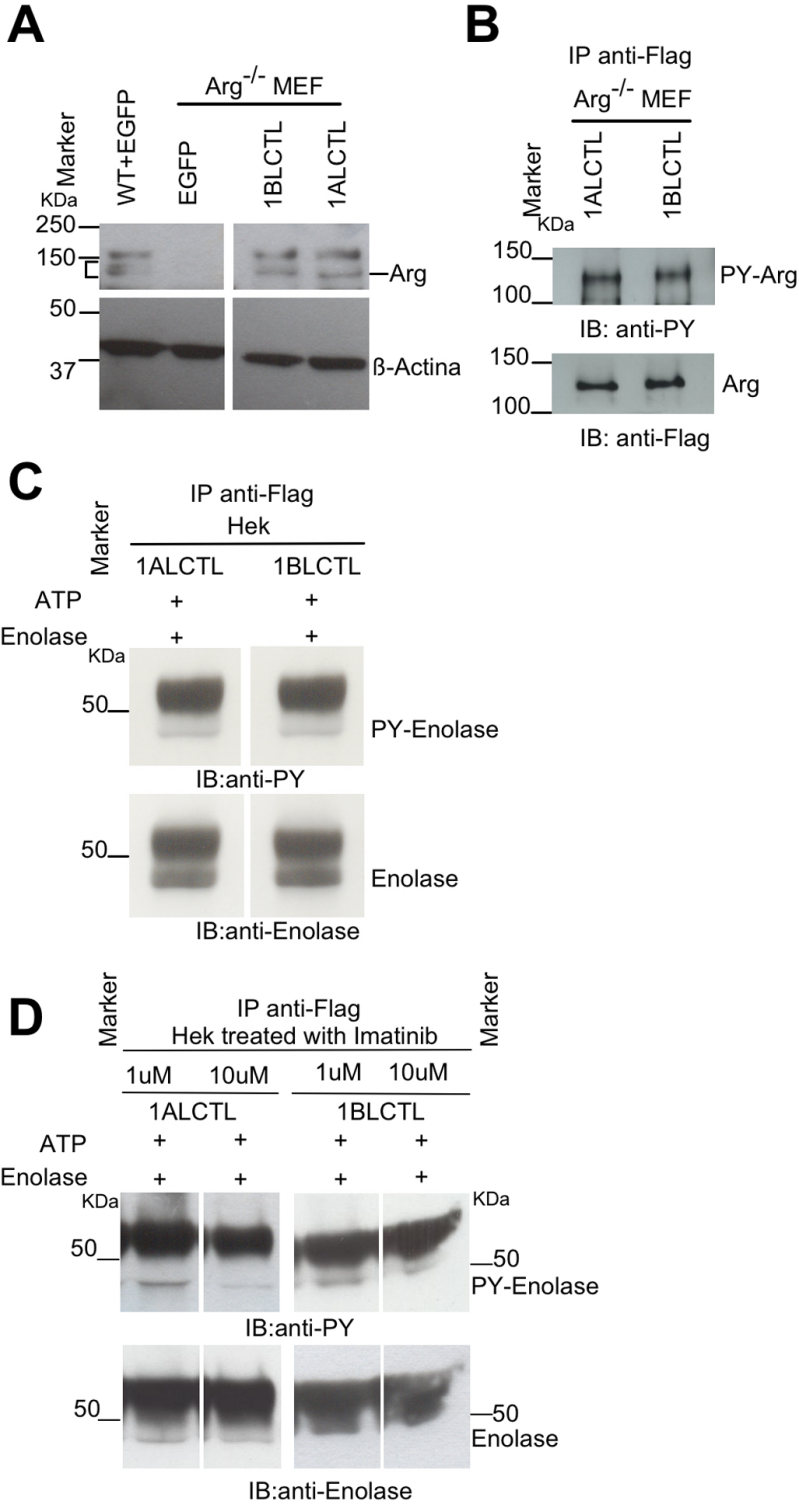
**Stable transfected Arg isoforms and their kinase activity.** (A) Western blots of lysates of wt MEF, Arg^−/−^ MEF transfected with empty vector (EGFP) and Arg^−/−^ MEF transfected with1ALCTL or 1BLCTL isoforms. Blots were hybridised with antibodies against Arg and β-actin; endogenous (square bracket) and recombinant (dash). Arg bands are indicated. (B) Western blot of Arg^−/−^ MEF transfected with the indicated Arg isoforms, immunoprecipitated (IP) with antibody against Flag, blotted and hybridized (IB) with antibodies against phosphotyrosine (PY) and Flag. (C) Tyrosine kinase assay *in vitro* of the indicated Arg isoforms transfected in Hek cell line. (D) Tyrosine kinase assay *in vitro* of the indicated Arg isoforms transfected in Hek cell line cultured for 3 h in presence of Imatinib 1 µM or 10 µM. In C and D, the cellular lysates were IP with antibody against Flag. Kinase reaction of IP proteins was performed in presence of ATP and enolase. IB with antibodies against PY and enolase.

To study the role of 1ALCTL and 1BLCTL Arg isoforms in fibroblast activation, we cloned into the stable expression vector pCX-C1-EGFP plasmid (Cinti et al., 2015) the corresponding Arg cDNA sequences. The vectors containing the inserts 1ALCTL or 1BLCTL have been transfected in Arg^−/−^ MEF (indicated as 1ALCTL MEF and 1BLCTL MEF, respectively) and the EGFP empty vector has been transfected in wt and Arg^−/−^ MEF (indicated as wt MEF and Arg^−/−^ MEF, respectively). After 2–4 steps of purification by cell sorting, the percentage of EGFP positive cells was from 90 to 95% for all types of transfection (Fig. S1A). Western blot confirmed that 1ALCTL MEF and 1BLCTL MEF expressed the Flag-Arg proteins at the expected molecular weight and at the same level of wt MEF (1BLCTL/wt Arg was 1.06, 1ALCTL/wt Arg was 1.08) (Fig. 1A). The tyrosine kinase activity of the two isoforms has been evaluated with anti-phosphotyrosine antibody on the isoforms immunoprecipitated with anti-Flag antibody (Fig. 1B; Fig. S1C). We performed an *in vitro* kinase assay on Hek cells transfected with the two Arg isoforms and we showed that both immunoprecipitated isoforms were able to phosphorylate the enolase protein (Fig. 1C; Fig. S1B). In addition, the transfected Hek cells treated with Imatinib, an inhibitor of Arg tyrosine kinase activity, evidenced that the two isoforms were sensitive to the drug, in particular to Imatinib concentration of 10 µM (Fig. 1D).

### The 1ALCTL and 1BLCTL Arg isoforms are differently able to activate Arg^−/−^ MEF

**Fig. 2.**
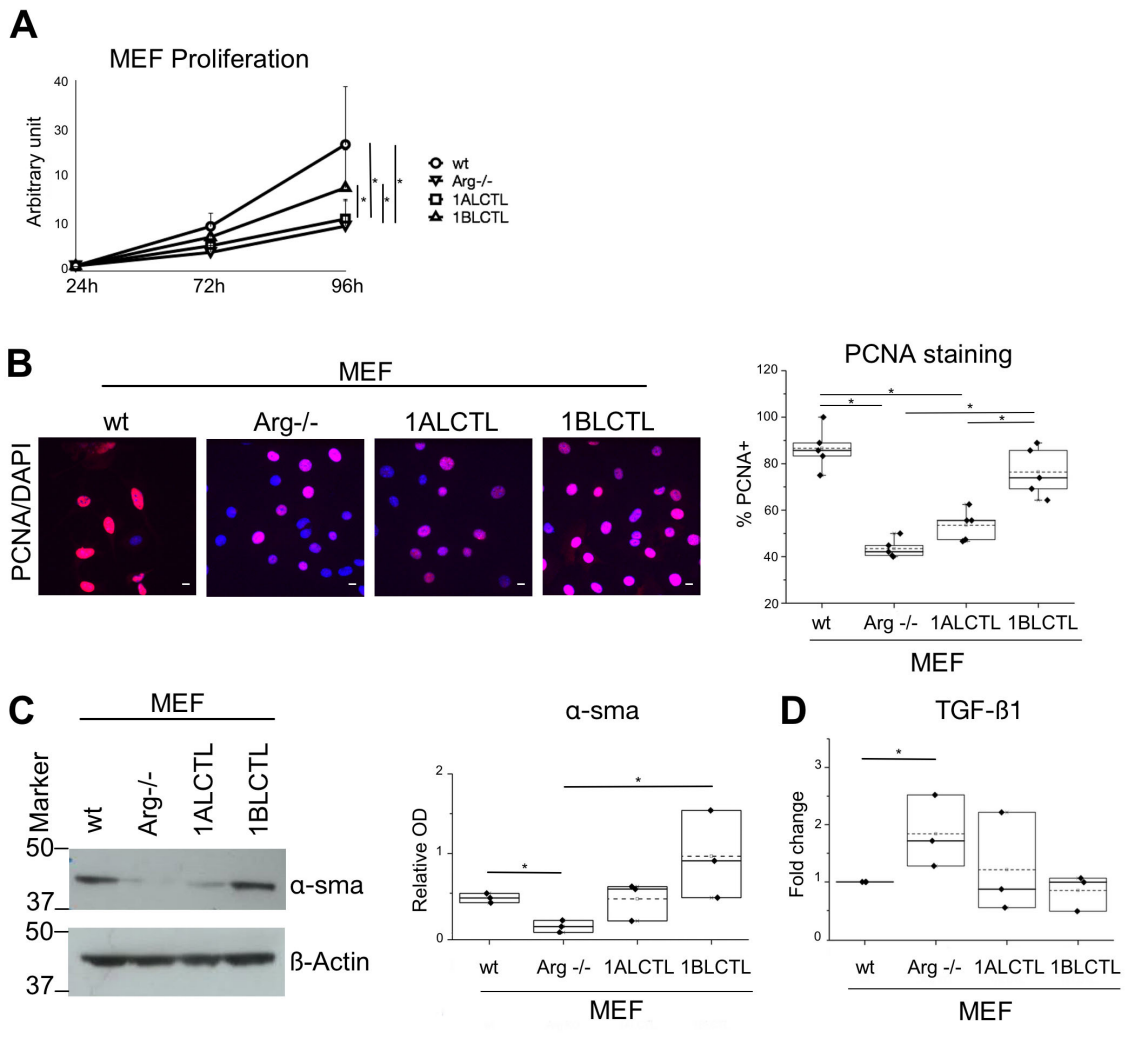
**Proliferation and activation of the different type of MEF.** (A) Growth curves of MEF, number of growing cells at 72 and 96 h with respect to 24 h of culture (*n*=5). (B) Images by confocal microscope of the indicated MEF stained with anti-PCNA antibody (red) and with DAPI (blue) after 24 h of culture. In the graph, the percentage of nuclear PCNA positive cells obtained counting 100 DAPI positive cells in fields randomly chosen. Dots represent the mean percentage of independent experiments (*n*=5). (C) Western blot of the indicated MEF hybridized with antibodies against α-sma and β-Actin. The box plot of normalized bands are shown (*n*=3). (D) TGFß1 transcript evaluated by real-time PCR in the indicated MEF. The values, calculated as 2^−ΔΔCt^, represented the fold change with respect to wt MEF chosen as calibrator sample (*n*=3).*P<0.05. Scale bars: 10 μm.

**Fig. 3.**
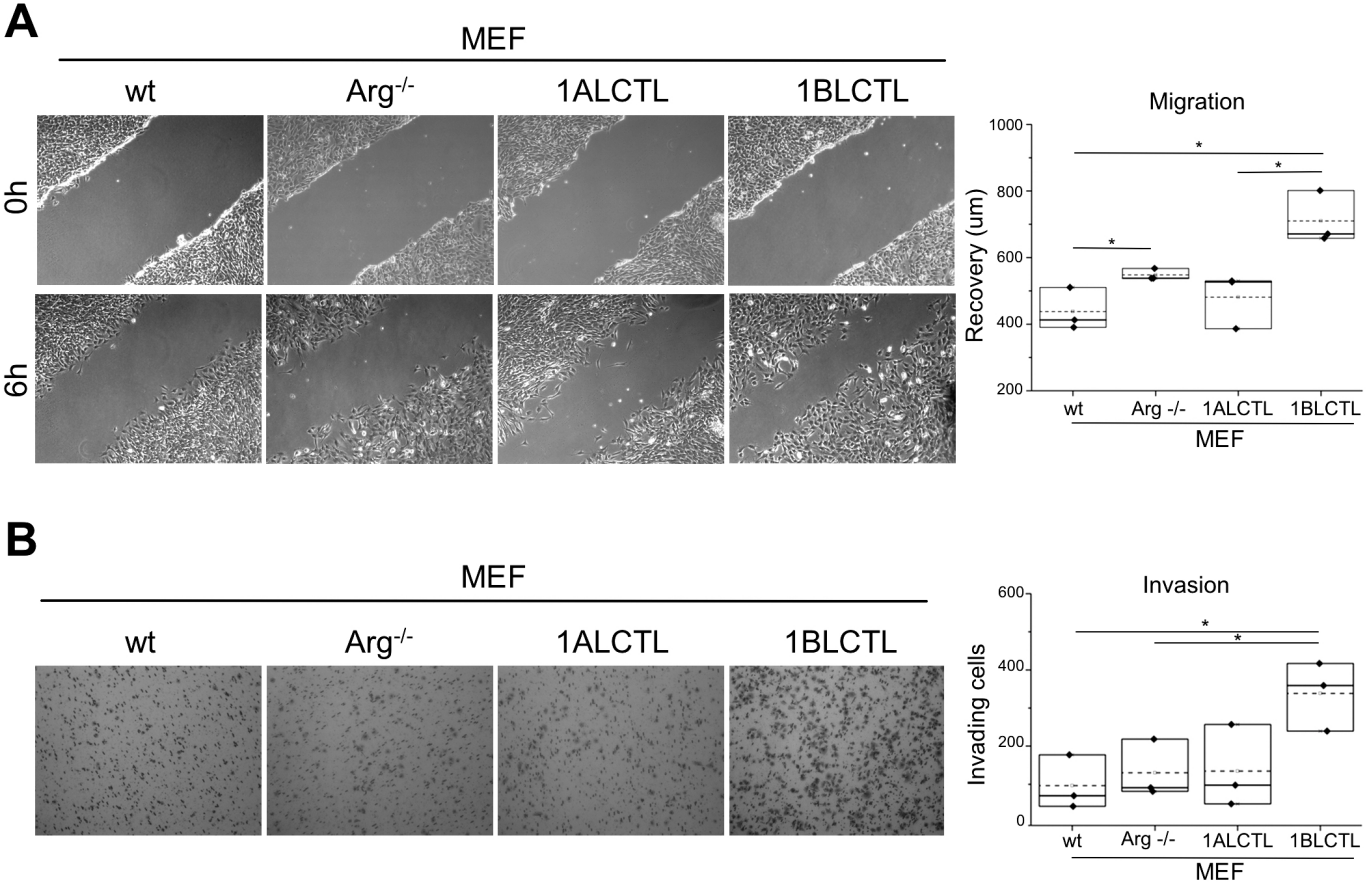
**Migration and invasion of transfected MEF.** (A) Wound healing assay at the scratch (0 h) and after 6 h of wound recovering, the graph represents the recovery expressed as µm in independent experiments (*n*=3). (B) Invading cells in Boyden chamber coated by collagen, the graph reports the mean number of invading cells counted in 10 randomly chosen fields for each sample in independent experiments (*n*=3). *P<0.05.

A characteristic of activated fibroblasts is the high proliferation rate (Barron and Rowley, 2012; Li et al., 2016), therefore, we evaluated the effect of Arg isoforms on MEF proliferation counting the viable cells at different time points. At 96 h the wt MEF were significantly more proliferating than Arg^−/−^ MEF. The 1ALCTL isoform maintained the MEF proliferation at the level of Arg^−/−^ MEF, while 1BLCTL induced a significantly higher proliferation activity than Arg^−/−^ and only slightly lower, in a non-significative manner, with respect to wt MEF (Fig. 2A). These data have been confirmed, evaluating by immunofluorescence the nuclear positivity of the proliferation markers PCNA (Fig. 2B). These findings highlighted the role of Arg, particularly of 1BLCTL, in fibroblast proliferation. An index of fibroblast activation, both in non-tumour myofibroblasts and in CAF, is the expression of α-sma (O'Connell et al., 2011). As shown, α-sma was expressed in wt MEF, while in Arg^−/−^ MEF it was almost undetectable. In 1BLCTL MEF α-sma was overexpressed with respect to Arg^−/−^ MEF, while in presence of 1ALCTL the expression mean value was level with wt MEF (Fig. 2C). Even the localisation of α-sma incorporated in stress fibres is a marker of activated fibroblasts (Goffin et al., 2006). The immunofluorescence evaluation showed that in Arg^−/−^ MEF and 1ALCTL MEF α-sma is diffusely localised in cytoplasm, while in wt MEF and 1BLCTL MEF the majority of α-sma colocalised with stress fibres (Fig. S2A). It is of note that the different MEF studied have a different capacity to produce TGFß1. In particular, the absence of Arg determined the increase of TGFß1 expression (Fig. 2D). The migratory ability of all MEF were analysed by wound healing and the wound recovery in Arg^−/−^ MEF significantly increased as compared to wt (Fig. 3A), confirming the already described inhibitory role of Arg on fibroblast migration (Peacock et al., 2007). Interestingly, in Arg^−/−^ MEF the migration ability increased further with respect to wt MEF after the transfection of 1BLCTL isoform, whose expression was at the same level of endogenous Arg in wt MEF (Fig. 3A). To test if Arg and its isoforms had a role in MEF invasiveness, we performed a collagen-based cell invasion assay. The invasiveness capacity determined by 1BLCTL is higher with respect to all the other cell types, which shared a similar invasive capacity (Fig. 3B). These data suggest that the Arg exon 1B and 1A differently modulate the migration and invasion of MEF.

### 1ALCTL and 1BLCTL Arg isoforms differently regulate cellular contractile structures

**Fig. 4.**
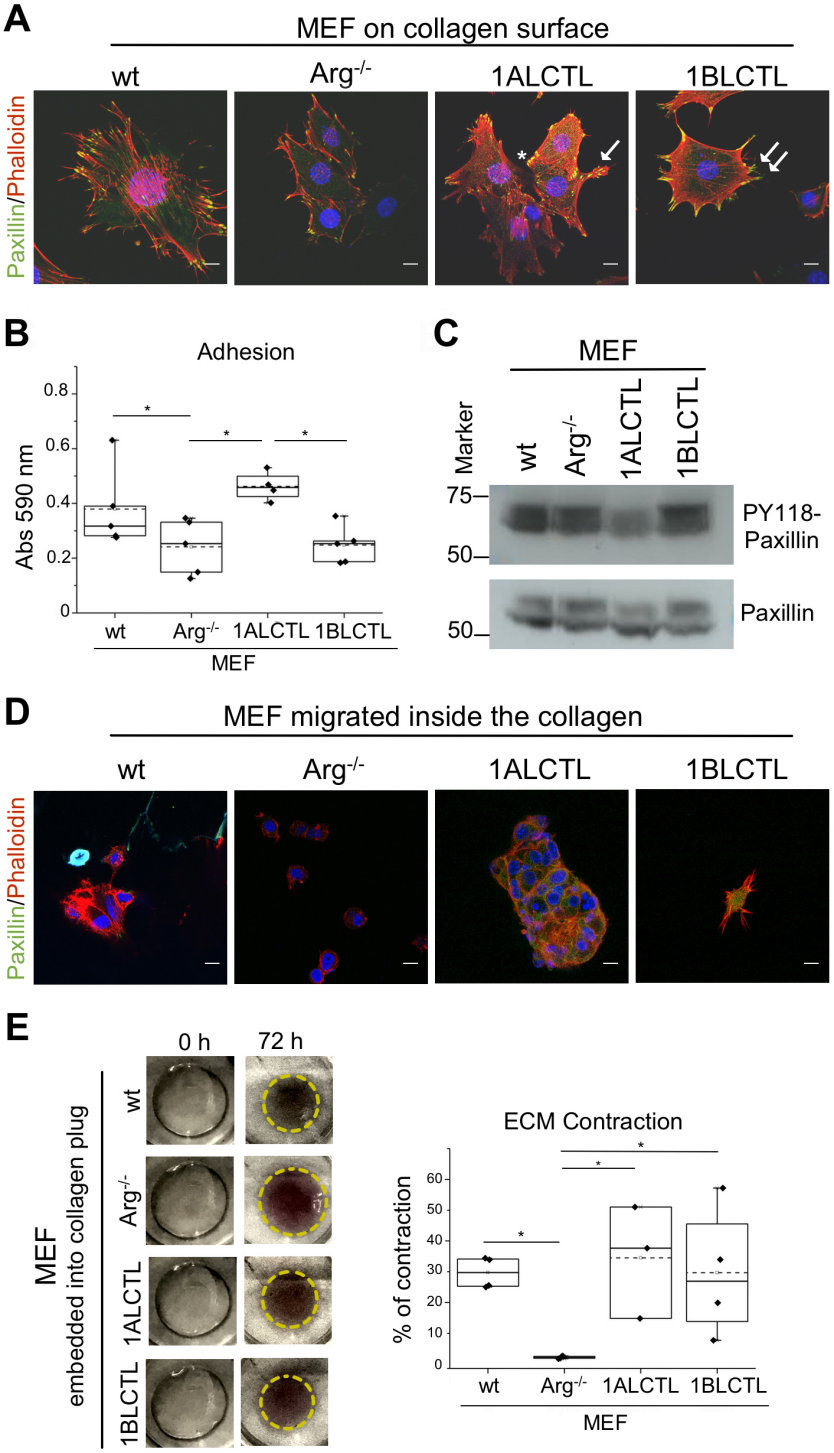
**Cytoskeletal components of transfected MEF and their interaction with collagen I.** (A) Distribution of paxillin (green) that represents focal adhesion and phalloidin (red) that represents stress fibres in the indicated MEF analysed on surface of collagen I gel after 4 h from plating. Nuclei were stained with DAPI (blue) and images were captured by confocal microscopy (63×). Asterisk, lamellipodia; arrow, retraction tail; double arrow, filopodia. (B) Graph of the adhesion ability of indicated MEF. The adhesion assay has been performed on 96-well collagen-coated plates and the values of absorbance measured at 590 nm represent independent experiments (*n*=5). (C) Western blot of indicated MEF hybridised with antibodies against phosphoY118-paxillin and paxillin. (D) Indicated MEF migrated for 4 h inside the collagen I gel, representing a 3D environment, and staining of paxillin (green) and phalloidin (red). Pictures have been captured by confocal microscopy (63×) in the z-stack range of 5–20 µm from the gel surface. (E) Collagen contraction by MEF embedded into collagen I gels. Areas of collagen I plugs with indicated embedded fibroblasts at zero (0 h) and 72 h of incubation. The graph shows the percentage of contraction induced by the MEF analysed in independent experiments (*n*=4). *P<0.05. Scale bars: 10 μm.

The cellular focal adhesions and stress fibres that are contractile structures essential for the force transmission between cells and matrix, which is a further characteristic of myofibroblast (Calvo et al., 2013), were evaluated. After plating MEF on collagen-coated glass for 4 h, we analysed by immunofluorescence the focal adhesion and stress fibre distribution in the cells. The wt MEF, grown on surface of collagen I gel as a 2D culture, showed a preferential peripheral distribution of the paxillin-positive focal adhesions and prominent phalloidin-positive stress fibres crossing the cytoplasm, in addition, wt MEF also had the highest cell spreading (Fig. S2B). Arg^−/−^ MEF were characterised by less evident focal adhesions and by thinner stress fibres that had a prevalent cortical distribution. 1ALCTL MEF had stress fibres crossing the cytoplasm and focal adhesions more evident in the retraction tail and in the opposite side of retraction tail (lamellipodia). 1BLCTL MEF maintained a cortical F-actin and a delocalisation of focal adhesions to filopodial structures (Fig. 4A). Given the morphology and the cell surface area of our MEF, we evaluated whether these features correlated to a specific adhesion ability. The cellular adhesion assay on collagen-coated wells showed that in Arg^–/–^ MEF the adhesion ability decreased significantly in respect to wt MEF. Only 1ALCTL isoform was able to restore it in Arg^–/–^ MEF at higher level than in wt MEF, although not significant (Fig. 4B). The adhesion ability of larger wt MEF is in accordance with literature data that correlate cell spreading with adhesion (Nardone et al., 2017). However, to explain why 1ALCTL MEF displayed an increased adhesion, despite a smaller spreading respect to wt MEF (Fig. S2B), we analysed phospho-Y118 paxillin. As shown in Fig. 4C, 1ALCTL MEF had a decreased phosphorylation of paxillin-Y118 with respect to all the other cellular types analysed and this result may justify the increased cellular adhesion of 1ALCTL MEF (Fig. 4B). In fact, it is known that phosphorylation of paxillin-Y118 leads to a focal adhesion disassembly and activity reduction (Coló et al., 2012). We also evaluated our cells’ migration for 4 h inside the collagen gel representing a 3D environment (Fig. 4D), as documented by the confocal microscope orthogonal views (Fig. S2D). In Fig. 4D the migrated wt MEF maintained phalloidin staining across the cytoplasm. Arg^−/−^ MEF had a more spheroid shape without a typical F-actin organisation. 1ALCTL and wt MEF inside the collagen were associated in groups of cells, 1BLCTL MEF had filopodia structures. 1ALCTL and 1BLCTL showed diffuse paxillin staining, but at 4 h of migration they did not show the focal adhesion organisation. Finally, the different MEF types were analysed for their ability to shrink the collagen plug in which they had been embedded for 72 h. The Arg^−/−^ MEF lost the ability to contract the collagen matrix. Both 1ALCTL and 1BLCTL restored the contraction to wt values (Fig. 4E). These data proved that Arg is essential for collagen-ECM contraction by fibroblasts.

### Wt and Arg^−/−^ transfected MEF generate ECM, which are architecturally and functionally different

**Fig. 5.**
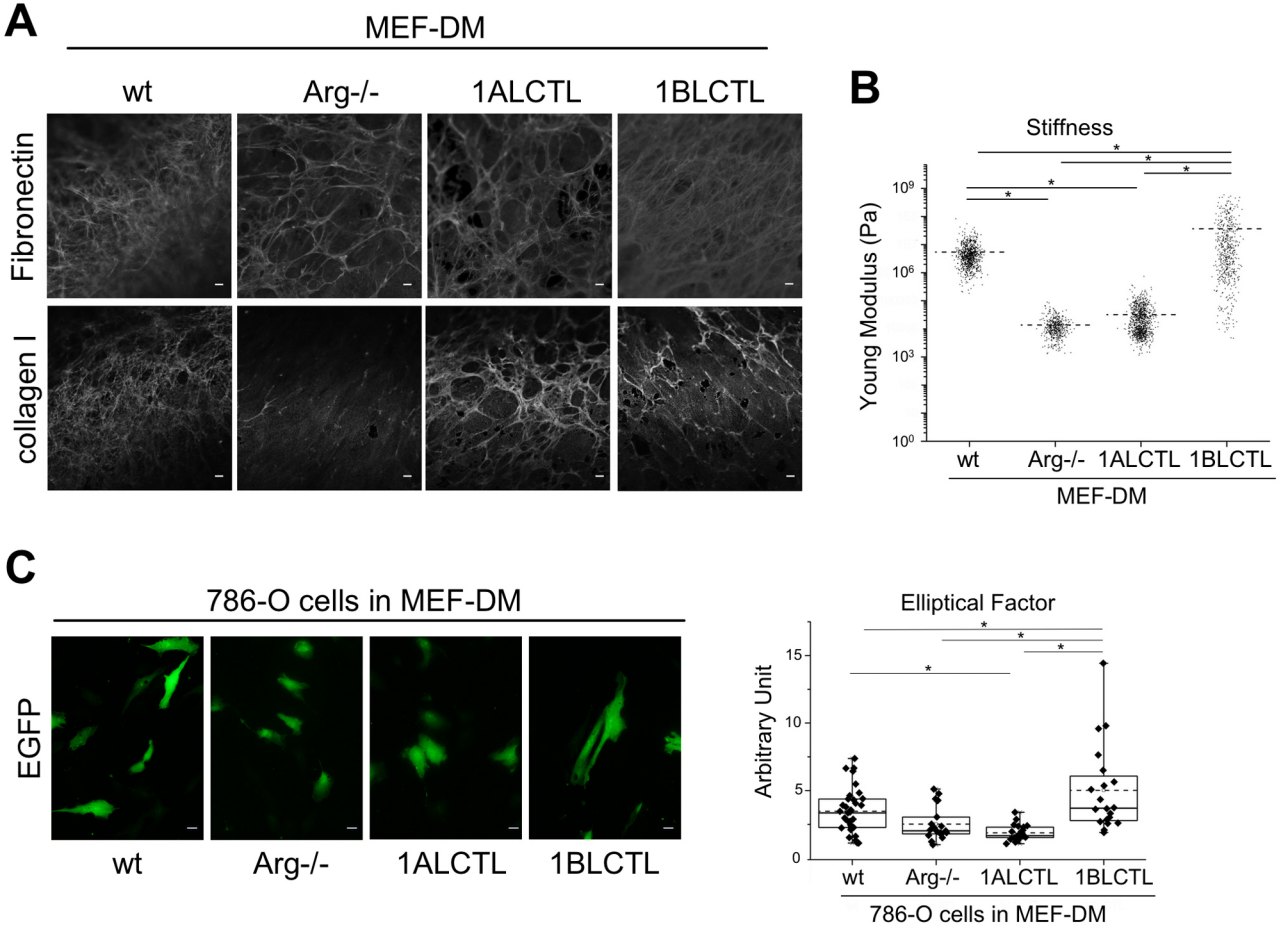
**Architecture and functionality of ECM produced by different MEF.** (A) Fibronectin and collagen I staining of MEF-derived matrices (MEF-DM) after the removal of MEF. (B) Evaluation of stiffness of the MEF-DM. Dot plot shows the stiffness expressed as Young modulus (Pa) of the different matrices, each dot represents a measure performed in different positions of MEF-DM during independent experiments (*n*=3). (C) 786-O cells transfected with pCX-C1-EGFP vector and grown on the indicated MEF-DM for 48 h, the graph shows the elliptical factor (ratio of the cell length/cell breadth) of 786-O cells evaluated in independent experiments (*n*=3). *P<0.05. Scale bars: 10 μm.

To test whether Arg and its isoforms had the ability to influence the production of stroma with different characteristics, we allowed the different MEF cells to produce and remodel their matrix for 10 days. Immunofluorescence staining was performed after the complete removal of MEF cells. The wt and 1BLCTL MEF produced bundles of fibronectin morphologically denser and more uniform than those produced by Arg^−/−^ and 1ALCTL MEF, in which the fibronectin frameworks appeared with larger mesh size. The staining of collagen I evidenced that Arg^−/−^ MEF had an undetectable deposition of the protein as compared to wt MEF, while the two transfected Arg isoforms enabled MEF to deposit collagen I (Fig. 5A). As fibronectin and collagen I deposition is relevant in determining matrix stiffness, a peculiar characteristic of fibrosis and tumour stroma (Bordeleau et al., 2015; Calvo et al., 2013), we assessed the relation between the newly produced ECM and their respective stiffness after removing MEF cells. Fig. 5B showed a decreased stiffness of ECM produced by Arg^−/−^ MEF with respect to wt MEF. The 1ALCTL Arg isoform was unable to elicit the production of matrix with stiffness at the level of wt MEF, while 1BLCTL transfection elicited the production of ECM with the highest stiffness. Finally, we investigated whether these differently Arg-modulated matrices were able to influence the cell morphology of invading tumour cells. As spindle-shape cells are associated to a more invasive phenotype (Tanaka et al., 2016), we evaluated the elliptical factor (EF) of 786-O RCC cells plated on ECM produced by our different MEF. The highest EF was observed in 786-O cells grown in the stiffest matrices produced by wt and 1BLCTL MEF, while in those produced by Arg^−/−^ and 1ALCTL MEF the tumour cells maintained a cobblestone shape (Fig. 5C). These results showed that the lack of Arg altered the ECM structure in terms of fibronectin, collagen I deposition and stiffness, and that the MEF expressing the 1BLCTL Arg isoform was able to produce ECM that enhance the elongation of the tumour cells.

## DISCUSSION

During the fibrosis process myofibroblasts, the activated form of fibroblasts continue to produce ECM resulting in abnormal scar tissue deposition. Myofibroblasts also occur in the context of growing tumour, and in some solid tumours they can generate a very abundant fibrotic stroma. The increase of the proliferation rate, the expression of several markers, the ECM production and remodelling are features of myofibroblasts (Yazdani et al., 2017) The knowledge of molecular players involved in fibroblast activation is important for the development of novel therapeutic strategies against fibrosis and growing tumours. Our data evidence the role of Arg in fibroblast activation. We previously showed that the 1ALCTL and 1BLCTL Arg isoforms determine some morphological and molecular changes in COS-7 cells (Bianchi et al., 2013).

Now, we have shown that Arg is necessary for fibroblast proliferation. Considering our two transfected isoforms, which maintained their tyrosine kinase activity (Fig. 1), the 1BLCTL isoform alone is sufficient to restore the proliferation rate of wt MEF (Fig. 2A,B). However, literature data show that Arg knockdown in MDA-MB-231, a breast tumour cell line, improves the proliferation of tumour epithelial cells (Gil-Henn et al., 2013), highlighting that the role of Arg in cellular proliferation could be cell specific. Numerous papers report the expression and localisation of α-sma as common markers of activated fibroblasts (O'Connell et al., 2011; Shi et al., 2016; Goffin et al., 2006). Our data showed that 1BLCTL isoform is able to restore both the α-sma expression and colocalization with stress fibres, negligible in Arg^−/−^ MEF (Fig. 2C; Fig. S2A). This finding suggests that Arg is necessary for fibroblast activation and that the fibrotic or the primary tumour stroma could be characterised by 1BLCTL Arg upregulation. Also an intriguing finding, in Arg^−/−^ MEF, showed us that TGFß1 expression was inversely related to Arg expression (Fig. 2D). We also described this interesting inverse relation between Arg and TGFß1 in renal tubular cells after Arg silencing (Torsello et al., 2016). It seems that the absence of Arg make the fibroblasts less responsive to the proliferative and activating effects of TGFß1 signals, suggesting that the TGFβ1 signalling is less efficient in absence of Arg, therefore, TGFβ1 production is upregulated. The TGFß1-mediated effects, carried out in wt MEF, were restored by 1BLCTL but not completely by 1ALCTL isoforms (Fig. 2A–C). A defined Arg isoform expression pattern may also be relevant for the movement ability of myofibroblasts in both tumour and fibrotic milieu. In accordance with this hypothesis, the wound recovery and the invasion of collagen matrices revealed that 1BLCTL isoform sustains this ability at the highest level in comparison with all the other MEF analysed. In particular, it seems that the Arg 1BLCTL protein level into the cell can modulate the migration. In fact, 1BLCTL, overexpressed either in Arg-endogenous-expressing or in Arg^−/−^ fibroblasts, determines a decrement of migration ability (Peacock et al., 2007; Bianchi et al., 2013). However, when in Arg^−/−^ cells the expression of recombinant Arg is only twofold over the endogenous Arg level the cells recover the migration abilty of wt cells (Peacock et al., 2007). In our transfected 1BLCTL MEF, in which the recombinant Arg is at the same level of wt MEF, their migration is higher than wt MEF. Instead, the 1ALCTL MEF were not able to reach the migration and invasion capacity shown by 1BLCTL MEF and an inhibitory activity of 1ALCTL cannot be excluded (Fig. 3A,B). The 4-h cultures evaluated in 2D or 3D environment of the different MEF could help to explain their behaviour. In fact, the highest spreading of wt MEF in 2D (Fig. S2B) may justify their adhesion capacity (Fig. 4B) (Nardone et al., 2017), despite the increased phosphorylation of Y118-paxillin. Instead, in 1ALCTL MEF the inhibition of paxillin phosphorylation, seems to account for the increment of cell adhesion. In 2D culture the decreased adhesion ability of 1BLCTL MEF (Fig. 4B) could contribute to a faster migration as it happens in Arg^−/−^ MEF (Fig. 3A). In the 3D environment of collagen matrix, 1BLCTL MEF are present as single cells (Fig. 4D) and this 3D-organisation associated with the decreased adhesion ability (Fig. 4B) can justify their high invasion capacity. 1ALCTL and wt MEF in addition to higher adhesion were also grouped together and this conformation could have delayed their invasion ability (Wong et al., 2014). In 3D culture the different spatial organization of cells can be due even to different modulation of adherent junctions. However, preliminary data in our different MEF (not shown) did not evidence significant differences in N-Cadherin expression, a marker of cell–cell adhesion in fibroblasts (Labernadie et al., 2017) and other cell–cell adhesion molecules need to be evaluated.

The ECM-contraction ability, another feature of myofibroblasts (Calvo et al., 2013), demonstrated that Arg is essential for collagen I contraction and that both 1ALCTL and 1BLCTL Arg isoforms are able, after 72 h, to restore the wt MEF contraction ability (Fig. 4E). An efficient ECM-contraction is due to working stress fibres and focal adhesions as well as to α-sma expression and incorporation in intracellular stress fibres (Calvo et al., 2013; Shinde et al., 2017; Shuttleworth et al., 2018). Our 1ALCTL and 1BLCTL MEF grown for 4 h inside collagen showed no focal adhesions (Fig. 4D). Otherwise, no difference in plug contractions was observed after 24 h (not shown). However, after leaving the 3D culture for 72 h the focal adhesions could have taken place, giving rise to plug contraction. It has to be noted that the dynamics of focal adhesions seems to be different in a 2D culture compared to a 3D culture (Chiu et al., 2014). Moreover, the α-sma expression (Fig. 2C) with its incorporation in stress fibres (Fig. S2A) may counteract the lack of functional focal adhesion preserving cell contractility.

The MEF analysed in this study were able to secrete and organise fibronectin and collagen I differently (Fig. 5). It is of note that Arg^−/−^ MEF failed to deposit the collagen I, which is currently deposited by myofibroblasts during fibrosis and tumour progression (Karsdal et al., 2017). This finding underlines the unique role of Arg in the production of collagen matrix. Otherwise, 1BLCTL MEF produced networks of collagen I and fibronectin that are similar to those produced by wt MEF and this condition correlated with the highest stiffness of ECM, measured after removal of MEF. The ECM stiffness is due to the amount of collagen and fibronectin fibres, their cross-link and ECM morphology (Di Stefano et al., 2016; Mierke et al., 2017). The specific role of Arg in producing fibronectin and collagen matrix turned out to be also significant in producing a specific matrix framework able to modulate the tumour cell morphology. In fact, the highest elliptical factor was induced in 786-O RCC cells when they grew in the high-stiffness-ECM produced by wt and 1BLCTL MEF (Fig. 5B,C). This finding is particularly relevant since it has been described that a fabricated matrix when forces the cells to assume an elongated morphology becomes able to select the cells with a more aggressive behaviour (Mazzini et al., 2015). In fact, the elongated morphology of tumour cells reveals a more invasive phenotype (Tanaka et al., 2016).

In conclusion, Arg isoform 1BLCTL has a major role in proliferation, migration/invasion of fibroblasts and in inducing a milieu able to modulate tumour cell morphology, while 1ALCTL isoform has a role in MEF adhesion maintaining active focal adhesions. On the whole, the presence of Arg in MEF supports the proliferation, activation, adhesion, ECM contraction and stiffness, while the absence of Arg affected these myofibroblast features.

## MATERIALS AND METHODS

### Molecular cloning of human Arg isoforms 1BLCTL and 1ALCTL

Full-length human Arg 1BLCTL and 1ASCTL cDNA maintaining the in-frame FLAG sequence, were excised from pFLAG-CMV2 vectors (Bianchi et al., 2008) and cloned into a pCX-C1-EGFP plasmid (Cinti et al., 2015). After cloning, the 1ALCTL cDNA has been obtained by inserting the Arg exon II in 1ASCTL using a QuickChange site-directed mutagenesis assay (Stratagene) as described in Bianchi et al. (2013). Restriction and sequencing analyses were performed on all the intermediate and also in the final constructs. Empty pCX-C1-EGFP plasmid was used as negative control vector for mock transfections.

### Cell culture and transfection

Wild-type (wt) or Arg^−/−^ MEF (Koleske et al., 1998) were grown in Dulbecco's minimum essential medium (DMEM) supplemented with 10% FBS, 1% of Pen/Strep, Fungizone and Glutamine (Euroclone), at 37°C and 5% CO_2_. These cells were split and plated to reach a 70–80% confluence on the day of transfection. pCX-1ALCTL-EGFP, pCX-1BLCTL-EGFP and empty vector plasmids were alternatively transfected into Arg^−/−^ MEF and the empty vector also into wt MEF by electroporation using Neon Transfection System (Life Technologies) according to the manufacturer's instructions. Transfected cells, resuspended in growth medium without antibiotics were plated and after 24 h transferred in growth medium containing 1 mg/ml of G418 (Sigma-Aldrich) for a 10 day selection. Transfected cells were stained by Propidium Iodide solution (Biolegend) to exclude dead cells and sorted on the basis of EGFP expression using MOFLO Astrios Cell Sorter and analysed by Kaluza software (Beckman Coulter). All the transfected cells were sorted to reach a EGFP purification level from 90 to 95%.

The human embryonic kidney Hek cell line, and the 786-O renal cell carcinoma cell line (ATCC) have been cultured in DMEM supplemented with 10% FBS (Euroclone), at 37°C and 5% CO_2_. These cell types (75×10^4^ cells) have been transfected using the vectors pCX-1ALCTL-EGFP, pCX-1BLCTL-EGFP in the Hek cells, pCX-C1-EGFP in 786-O cells using Lipofectamine 3000 Reagent (Invitrogen) following the manufacturer's instructions. The transfected Hek cells, when requested, were cultured for 3 h in presence of 1 µM or 10 µM Imatinib mesylate as Arg tyrosine kinase inhibitor (Cayman Chemicals).

### Western blot

Cell lysates were prepared, separated by SDS Nupage 4–12% and blotted on nitrocellulose membrane (all Life Technologies) as described (Cifola et al., 2011). The protein standard Dual Color Marker loaded on the gel were from Bio-Rad. The blotted membranes were probed with antibodies against: Flag (1:1000, Sigma-Aldrich), GFP (1:1000, Invitrogen), Arg (1:400, Millipore), PhoshoY (4G10, 1:1000, Millipore), α-sma (1:1000, Dako), PhosphoY118-Paxillin (1:1000, Cell Signaling Boston), paxillin (1:1000, Cell Signaling). ECL (Pierce, Thermo Fisher Scientific) detected the antigen-antibody complexes. Densitometry of the bands was analysed by ImageJ software (NIH).

### Tyrosine kinase activity

The tyrosine kinase activity of 1ALCTL and 1BLCTL isoforms has been assessed as described (Spirli et al., 2012) on the immunoprecipitated protein using antibody against Flag (10 µg/ml) as described (Torsello et al., 2016) and evaluating the autophosphorylation of Arg isoforms transfected in Arg^−/−^ MEF. The ability to tyrosine phosphorylate the enolase substrate (Sigma-Aldrich) has been evaluated by an *in vitro* kinase assay on the immunoprecipitated Arg isoforms transfected in Hek cells (0.5 mg of cell lysates). The reaction has been performed in 20 µl of the following solution: 16 µl of 50 mM MgCl2, 2 µl of 1 mM ATP (Sigma-Aldrich) and 2 µg of enolase. After 30 min of incubation at 30°C, the reaction was stopped by adding SDS sample buffer. The reaction product was blotted and probed with antibodies against anti-PhosphoY and anti-enolase (Santa Cruz Biotechnology Heidelberg) and visualised by the ECL system.

### Immunofluorescence

MEF cells (1×10^5^) were seeded for 4 h on glass coverslips coated with collagen I Rat tail (Gibco, Life Technologies), fixed and incubated with antibody against paxillin (1:50, Becton Dickinson) to evidence focal adhesion or against α-sma (1:50, Abcam). Stress fibres have been labelled by Alexa 594-phalloidin (1:100, Molecular Probes Invitrogen) and nuclei were counterstained with Mounting DAPI (Molecular Probes Invitrogen). The cell surface area was evaluated as described (Bianchi et al., 2013) in EGFP positive cells plated as above. MEF cells proliferating for 24 h on glass coverslips were stained with PCNA (1:50, Clone PC10, Santa Cruz Biotechnology). Immunofluorescence pictures were obtained with confocal microscope Zeiss LSM710, using 63× or 40× objectives, equipped with Zen2009 software (Zeiss).

### Real-time quantitative PCR

Total RNA extraction and reverse transcription were performed as described (Bianchi et al., 2008). Real-time quantitative PCR was carried out with a TaqMan Gene Expression Assay (Applied Biosystems) according to manufacturer's instructions, using commercial kits (TGFß1 Hs00998133_m1 Human; GAPDH Hs99998805_m1 Human, both Applied Biosystem). The relative levels of the different transcripts were calculated as 2^−ΔΔCt^ that represented the fold change with respect to the calibrator sample considered equal to 1.

### Cell proliferation

Cell proliferation was monitored by Trypan Blue exclusion counting living cells. MEF were seeded at 50×10^4^/60 mm dish and trypsinized after 24, 72 and 96 h of culture. The proliferating cells were also monitored at 24 h evaluating the percentage on nuclear positivity of PCNA marker on 100 DAPI positive cells analysed for each MEF type in randomly chosen fields of several independent experiments.

### Cell migration and invasion

Wound healing has been performed as described (Di Stefano et al., 2016). Monolayers of MEF cultures on six-well plates, were scratched with a pipette tip and photographed with a digital camera mounted on an inverted microscope Olympus (100× magnification). The cultures were photographed again after 6 h. Initial and final wound width was measured using *segmented lines* ImageJ software tool to track two segmented lines corresponding to the wound edges. *Save XY coordinates* tool was used to obtain the straight line equation by which the distance between the two straight lines corresponding to the edges of the wound was calculated. Wound recovery was calculated as a mean difference between initial and final wound width obtained in three different fields for each well of the same experiment. Invasion has been evaluated using QCM™ 24-Well Collagen-Based Cell Invasion Assay, the membrane of Boyden chamber was coated by manufacturer (Millipore) with a solution of 0.3% collagen, composed by type I (85%) and type III (15%) collagen from chicken. The manufacturer's instructions were followed and 60×10^3^ fibroblasts were plated on the upper chamber. After 3 h at 37°C, we stained and microphotographed the porous membrane. The migrated cells were counted by two blinded operators at 400× in 10 different fields randomly chosen for each sample in all independent experiments with ImageJ software.

### Cell adhesion and ECM contraction

Adhesion assay has been performed as described (Di Stefano et al., 2016). ECM contraction has been performed using 75×10^3^ fibroblasts embedded in 100 µl of collagen I rat tail. The mixture of collagen and cells was seeded on a 35-mm glass-bottom MetTek dish. Once the gels were set, photographs have been taken (t0) and gels containing fibroblasts were maintained in culture medium for 72 h (t72) when other photographs have been taken. To obtain the gel contraction value, the specific areas of the gel at t0 and t72 were measured using ImageJ software. The percentage of gel contraction of different independent experiments was calculated using the formula 100×[(gel area t0−gel area t72)/gel area t0].

### Cell-derived matrices and stiffness

Cell-derived matrices were obtained on coverslips prepared as described (Kaukonen et al., 2016). 1.5×10^5^ MEF of each type were respectively plated on coverslip. When the cells were confluent, ascorbic acid (50 µg/ml, Sigma-Aldrich) was added and the medium changed every 2 days for 10 days. Cells were then removed by extraction buffer (0.2% sodium deoxycholate in 10 mM Tris-HCl, pH 8.0, supplemented with protease inhibitors). Solubilized cellular material was then gently washed two to three times for 5 min each on ice with a washing buffer composed of 2 mM Tris-HCl, pH 8.0, supplemented with protease inhibitors. A subsequent treatment at 37°C with DNAse I (Sigma-Aldrich) for 20 min was performed to assure removal of DNA associated with nuclear debris (Hedman et al., 1979). These MEF-derived matrices (MEF-DM) have been stained with antibodies against Fibronectin (1:1000, Dako) or collagen I (1:500, Abcam). The stiffness of these matrices has been measured by Atomic Force Microscopy as described (Di Stefano et al., 2016). 15×10^4^ 786-O cells, transfected with the pCX-C1-EGFP vector, have been plated on each of these MEF-DM for 48 h. The EF of 786-O cells has been calculated as the cell length/breadth ratio by ImageJ software (NIH). Cell pictures have been taken with an Eclipse E800 microscope (Nikon) and LuciaG 5.0 software (Nikon) supported by a digital camera (Nikon, DS-U1). In three different independent experiments 20 to 40 cells have been analysed for each conditions.

### Statistical analysis

All molecular and functional effects of different cellular types were evaluated and/or quantified by two different operators blinded to experimental treatment. Differences between multiple groups were analysed using one-way ANOVA followed by post-hoc Tukey's test using OriginPro 2016 64BIT software. Values of *P*<0.05 were considered statistically significant. In the box/dot graphs showed, representing at least three independent experiments, the individual dot represents the single independent experiment, the boxes indicate the 25°–75° percentile, the continuous horizontal line into the box represents the median (**—**), while the dotted horizontal line represent the mean (- - -). Max and min (⊤ and ⊥) are indicated.

## Supplementary Material

Supplementary information
