# Correction: The 1ALCTL and 1BLCTL isoforms of Arg/Abl2 induce fibroblast activation and extra cellular matrix remodelling differently

**DOI:** 10.1242/bio.058751

**Published:** 2021-05-25

**Authors:** Barbara Torsello, Sofia De Marco, Silvia Bombelli, Elisa Chisci, Valeria Cassina, Roberta Corti, Davide Bernasconi, Roberto Giovannoni, Cristina Bianchi, Roberto A. Perego

There was an error in Biology Open (2019) 8, bio038554 (doi:10.1242/bio.038554).

The 1BLCTL PY-Enolase panel in Fig. 1C was inadvertently a duplicate of the 1ALCTL panel. The corrected and original panels are shown below; both the online full text and PDF versions of the paper have been corrected.

**Fig. 1C (corrected panel).**
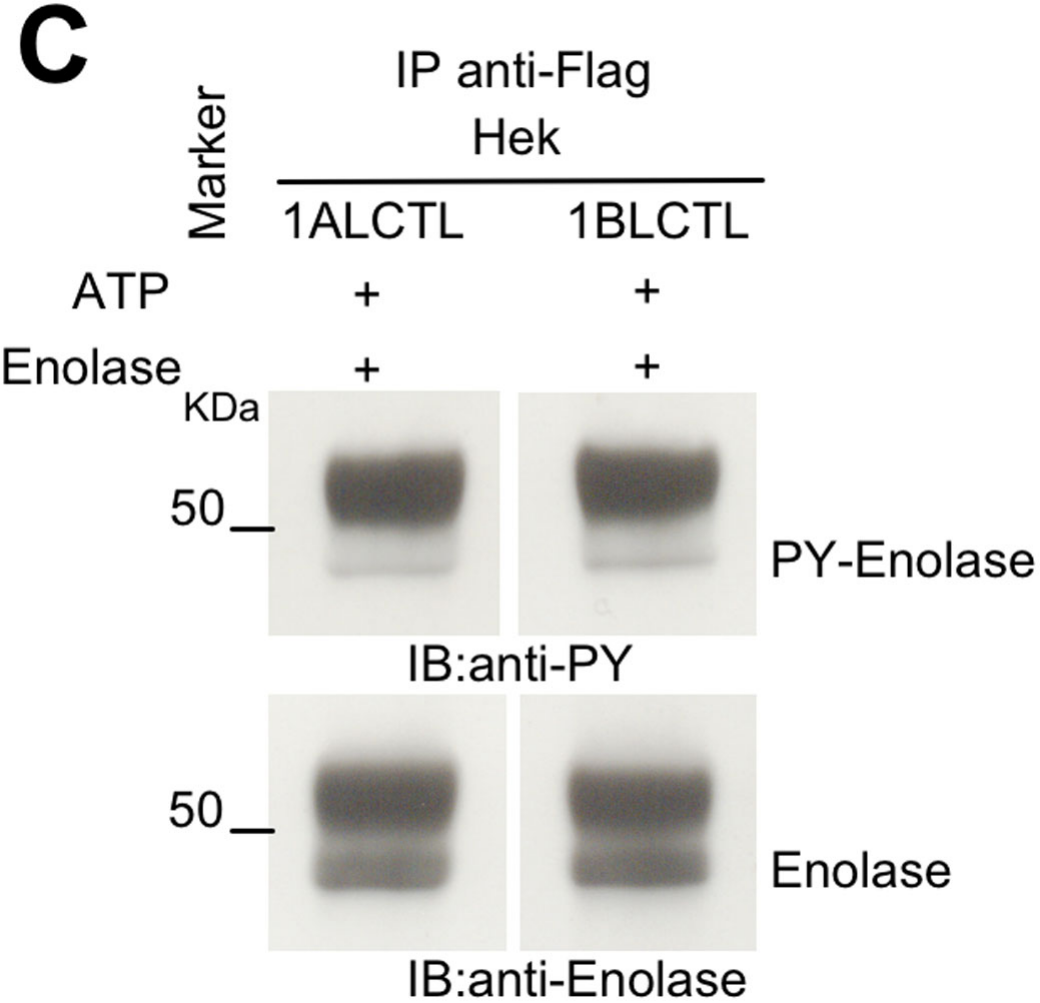
**Stable transfected Arg isoforms and their kinase activity.** (C) Tyrosine kinase assay in vitro of the indicated Arg isoforms transfected in Hek cell line. In C and D, the cellular lysates were IP with antibody against Flag. Kinase reaction of IP proteins was performed in presence of ATP and enolase. IB with antibodies against PY and enolase.

**Fig. 1C:**
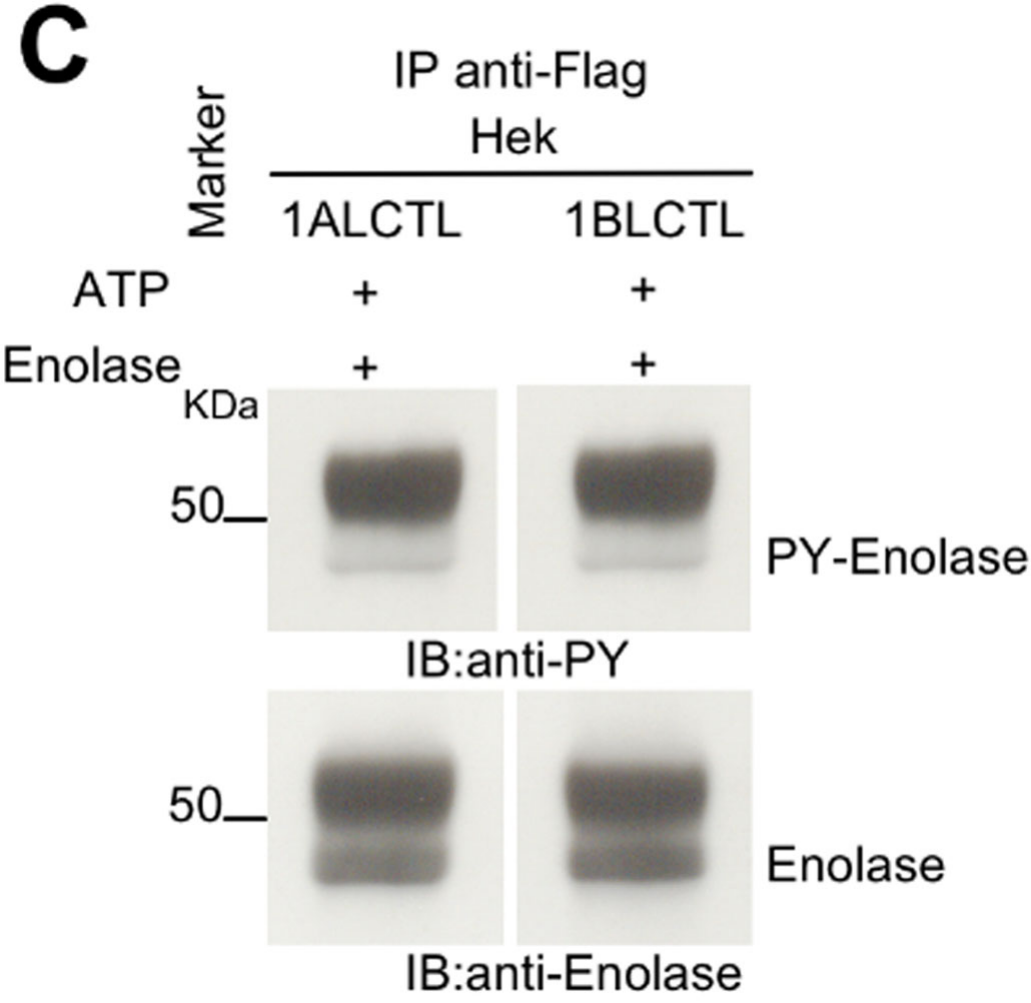
**(original panel). Stable transfected Arg isoforms and their kinase activity.** (C) Tyrosine kinase assay in vitro of the indicated Arg isoforms transfected in Hek cell line. In C and D, the cellular lysates were IP with antibody against Flag. Kinase reaction of IP proteins was performed in presence of ATP and enolase. IB with antibodies against PY and enolase.

The authors apologise to readers for this error, which does not impact the results or conclusions of the paper.

