# Supplementary material for: The 1ALCTL and 1BLCTL isoforms of Arg/Abl2 induce fibroblast activation and extra cellular matrix remodelling differently
Source: Biol Open. 2019 Mar 5;8(3):bio038554. doi: 10.1242/bio.038554 (PMC6451347; doi:10.1242/bio.038554)
Supplement: Supplementary information [file biolopen-8-038554-s1.pdf]

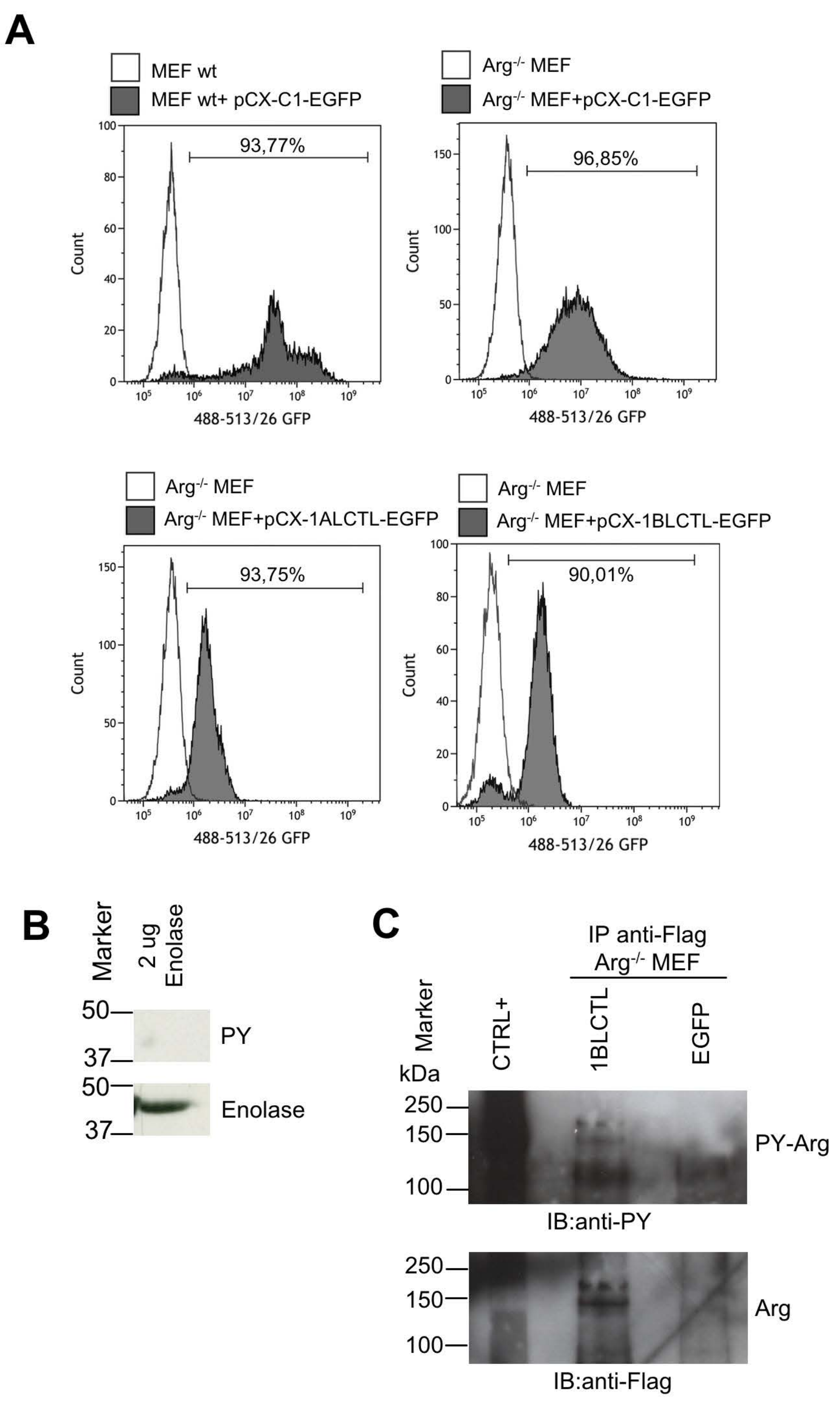

Figure S1. A: Percentage of recovering of EGFP positive cells of the indicated transfected MEF. B: Western blot of 2 µg of Enolase hybridized with antibodies against PhosphoTyrosine (PY) and Enolase. C: western blot of Arg$^{-/-}$ MEF transfected with the indicated vectors, immunoprecipitated (IP) with antibodies against Flag, blotted and hybridised (IB) with antibodies against Phosphotyrosine and Flag.

Biology Open • Supplementary information

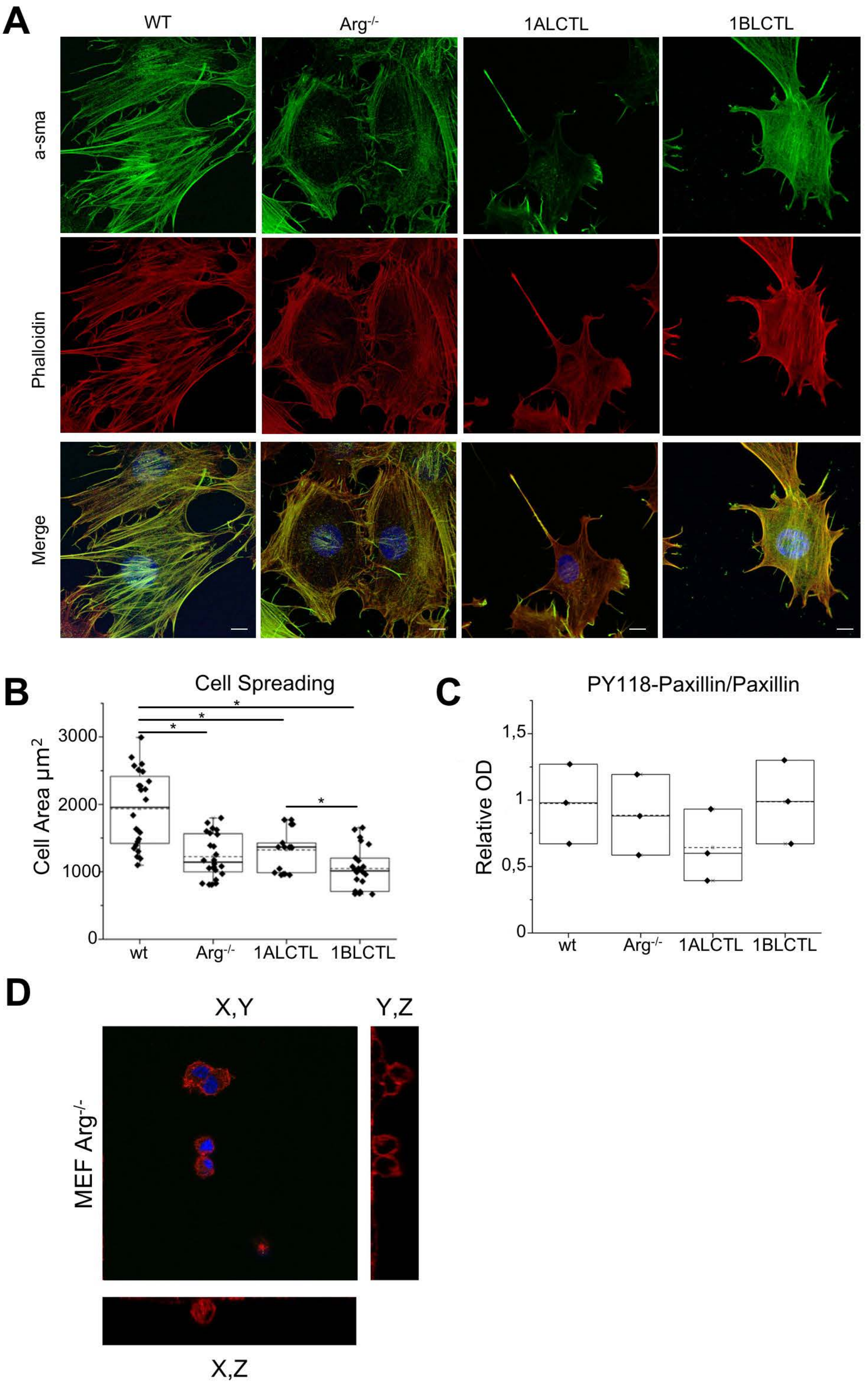

Figure S2. A: distribution of α-sma and stress fibres, respectively stained with antibody against α-sma (green) and phalloidin (red), in the indicated MEF. B: Dot blot of cell spreading of indicated MEF. The Cell Area expressed in µm has been measured by Image as described (Bianchi et al., 2013). Each dot represents ameasure of single cell area in independent experiments (n=3). *P<0.05.

Biology Open • Supplementary information

C: Dot plot of PY 118-Paxillin bands normalised for Paxillin (n=3). D: Representative image of transfected Arg$^{-/-}$ MEF migrated inside the collagen gel. Immunofluorescence staining of Phalloidin captured by Zeiss confocal microscopy. YZ and XZ sections have been obtained with ImageJ software (*Orthogonal Views Plugin*), after acquiring Z-Stack sections. 50 pictures along the Z-plane, 0.50 µm each, have been taken.
